# Supplementary material for: Ongoing brain state dynamics shape spontaneous awake reactivation and its relationship with memory
Source: bioRxiv. 2026 Sep 30:2026.09.24.754200. Preprint. [Version 2] doi: 10.64898/2026.09.24.754200 (PMC13648388; doi:10.64898/2026.09.24.754200)
Supplement: Supplementary file 1 [file NIHPP2026.09.24.754200v2-supplement-1.pdf]

bioRxiv preprint doi: https://doi.org/10.64898/2026.09.24.754200; this version posted September 30, 2026. The copyright holder for this preprint (which was not certified by peer review) is the author/funder, who has granted bioRxiv a license to display the preprint in perpetuity. It is made available under a CC-BY 4.0 International license.

Supplementary Results

TMS Modulation of Brain State-Memory Relationships during Reactivation

We further characterized whether relationships between brain state strength during reactivation and associative forgetting varied across TMS conditions. For each brain state, we asked whether associative forgetting was predicted by brain state strength during reactivation, TMS site (mean centered), and their interaction, followed by correlations within each TMS group. For State 2, consistent with the results across all participants, State 2 strength during reactivation was significantly related to forgetting when TMS site was included in the model (main effect: $b = 1.09 \pm .34$, $t = 3.20$, $p = .002$). The TMS × State 2 interaction was not significant ($b = –0.19 \pm .68$, $t = –0.29$, $p = .776$), indicating that the relationship between forgetting and State 2 strength during reactivation did not differ reliably across TMS sites. Indeed, relationships were in the same direction in both TMS conditions, with greater expression of the 2A pole during reactivation being associated with greater forgetting (Control TMS group, $r = 0.51$, $p = .008$; LOC TMS group, $r = 0.35$, $p = .083$; Fig. S1B).

A distinct pattern was observed for State 4. While the main effect of State 4 strength during reactivation was not significant when TMS site was modeled ($b = –.29 \pm .42$, $t = –0.70$, $p = .486$), the TMS × State 4 strength interaction was significant ($b = 2.07 \pm .84$, $t = 2.48$, $p = .017$), indicating that the relationships between State 4 strength during reactivation and forgetting differed between TMS sites. Specifically, this relationship emerged only in the control TMS group ($r = –0.49$, $p = .010$; Fig. S1B, top right) but was not reliable in the LOC TMS group ($r = 0.21$, $p = .305$; Fig. S1B, bottom right).

State 1 strength during reactivation was not significantly related to forgetting (main effect: $b = 0.83 \pm .47$, $t = 1.75$, $p = .086$; Control TMS group: $r = 0.19$, $p = .350$; LOC TMS group: $r = 0.29$, $p = .157$; Fig. S1B, left) and this relationship did not differ between TMS sites (interaction effect: $b = 0.66 \pm .95$, $t = 0.69$, $p = .491$), consistent with the prior analysis across all participants. For State 3, the main effect of state strength was not significantly related to forgetting (main effect: $b = 0.40 \pm .38$, $t = 1.06$, $p = .295$), but there was a marginal TMS × State 3 interaction ($b = –1.51 \pm .76$, $t = –1.99$, $p = .052$), indicating that the relationship between State 3 strength during reactivation and forgetting showed a trend-level difference across TMS sites. Greater expression of the 3A pole during reactivation was associated with increased forgetting in the Control TMS group ($r = 0.42$, $p = .035$), while this relationship was not reliable in the LOC TMS group ($r = –0.13$, $p = .528$; Fig. S1B, second right).

Together, these results indicate that the relationship between State 2 expression during reactivation and memory was relatively consistent across TMS sites, whereas the State 4 relationship differed across TMS sites and was only present in the Control TMS group; State 3 showed a similar but weaker pattern of TMS-dependent modulation. Thus, the relationship between brain state expression during reactivation and memory was selectively modulated by TMS, with one remaining stable (State 2) and other being altered by perturbation of post-encoding activity (States 3 and 4).

Brain States and Global Signal Dynamics Associated with LOC and Hippocampal Reactivation: Full Results

bioRxiv preprint doi: https://doi.org/10.64898/2026.09.24.754200; this version posted September 30, 2026. The copyright holder for this preprint (which was not certified by peer review) is the author/funder, who has granted bioRxiv a license to display the preprint in perpetuity. It is made available under a CC-BY 4.0 International license.

To assess whether global brain dynamics linked to coordinated reactivation events (reported in the Results) reflect properties specific to coordinated hippocampal-cortical co-reactivation, or whether they generalize to reactivation events identified in individual brain regions, we examined coupling with global brain dynamics separately for hippocampal and LOC reactivation events. This approach allowed us to test the prediction that reactivation propagates from the hippocampus outward to neocortical regions along the principal gradient (Kaefer et al., 2022). This framework predicts that distinct brain state profiles would be active during hippocampal vs. LOC reactivation events, such that hippocampal reactivation is linked with brain states reflecting the association pole of the S-A axis (3B), while LOC reactivation is linked with the sensorimotor pole of the S-A axis (3A).

Indeed, reactivation events in these two regions were associated with distinct brain states (Fig. S4A). A two-way repeated-measures ANOVA on the brain state strength measure revealed significant main effects of brain state ($F_{3,153} = 12.64$, $p < .001$) and reactivation region ($F_{1,51} = 8.87$, $p = .004$), as well as a significant interaction between the two factors ($F_{3,153} = 53.01$, $p < .001$). Consistent with the principal gradient prediction, State 3 strength significantly differed between hippocampal versus LOC reactivation (pairwise comparison: $t_{51} = –9.05$, $p_{FDR} < .001$). LOC reactivation events were coupled with the 3A brain state pole (one sample t test: $t_{51} = 9.98$, $p_{FDR} < .001$), indicating LOC reactivation is more closely aligned with the sensorimotor pole of the S-A axis (Margulies et al., 2016). By contrast, hippocampal reactivation tended to occur during the 3B brain state pole ($t_{51} = –2.32$, $p_{FDR} = .024$), reflecting the association pole of the S-A axis. In addition to State 3, State 1 strength was greater or biased towards the A pole during hippocampal compared to LOC reactivation (pairwise comparison: $t_{51} = 7.32$, $p_{FDR} < .001$). This indicates that hippocampal reactivation was more reliably coupled with an internally oriented brain activity pattern (1A, one sample t test: $t_{51} = 9.96$, $p_{FDR} < .001$), strongly weighting the DMN. In contrast, LOC reactivation was not reliably coupled with State 1 ($t_{51} = –1.21$, $p_{FDR} = .231$), suggesting that visual reactivation in isolation does not preferentially occur during internally or externally oriented states, instead being linked with activation of the broader visual network (evident in States 2 and State 3). In contrast to States 1 and 3, which were distinctly linked with hippocampal vs. LOC reactivation events, States 2 and 4 did not reliably differ in their coupling to hippocampal vs. LOC reactivation (pairwise comparisons: State 2: $t_{51} = –0.55$, $p_{FDR} = .587$; State 4: $t_{51} = 0.06$, $p_{FDR} = .952$), suggesting that coupling of coordinated hippocampal-LOC reactivation with States 2 and 4 reflects a shared property, rather than being driven by a specific region. Together, these findings align with predictions of the gradient cascade model of reactivation (Kaefer et al., 2022) and additionally reveal that hippocampal reactivation is more selectively tied to an internally oriented processing state, suggesting that the conditions supporting hippocampal reactivation may be more constrained than those supporting sensory cortical reactivation.

Analysis of the GS showed that reactivation events in the two regions differed in their preferred GS phase (Kuiper two-sample test: $V = 0.072$, $p < .001$; Fig. S4B). While hippocampal reactivation events were associated with the GS peak (Rayleigh test: $p < .001$), consistent with coordinated events (Fig. 4B), LOC events were linked with earlier GS phases, peaking around –45° (Rayleigh test: $p < .001$; Fig. S4B). This observation of a phase offset, with LOC reactivation preceding hippocampal reactivation in the GS phase, appears inconsistent with the gradient cascade model, which predicts that hippocampal reactivation would precede cortical reactivation.

Lastly, we asked whether LOC and hippocampal reactivation events occurred more frequently during minute-long time windows with high GS amplitude. Indeed, analogous to coordinated reactivation events, reactivation rate in each region was positively correlated with GS amplitude across time windows (i.e., Spearman correlation greater than 0; one-sample t-tests: LOC: $t_{51} = 3.92$, $p < .001$; hippocampus: $t_{51} = 8.84$,

bioRxiv preprint doi: https://doi.org/10.64898/2026.09.24.754200; this version posted September 30, 2026. The copyright holder for this preprint (which was not certified by peer review) is the author/funder, who has granted bioRxiv a license to display the preprint in perpetuity. It is made available under a CC-BY 4.0 International license.

$p < .001$). However, this link between reactivation and GS amplitude was significantly stronger for hippocampal reactivation (paired t-test: $t_{51} = 3.54$, $p < .001$; Fig. S4C). This observation of stronger coupling between hippocampal reactivation and GS amplitude, combined with differential locking of hippocampal reactivation to the peak GS phase (reported above), suggests a tighter association between hippocampal reactivation and GS fluctuations linked to lower arousal.

bioRxiv preprint doi: https://doi.org/10.64898/2026.09.24.754200; this version posted September 30, 2026. The copyright holder for this preprint (which was not certified by peer review) is the author/funder, who has granted bioRxiv a license to display the preprint in perpetuity. It is made available under a CC-BY 4.0 International license.

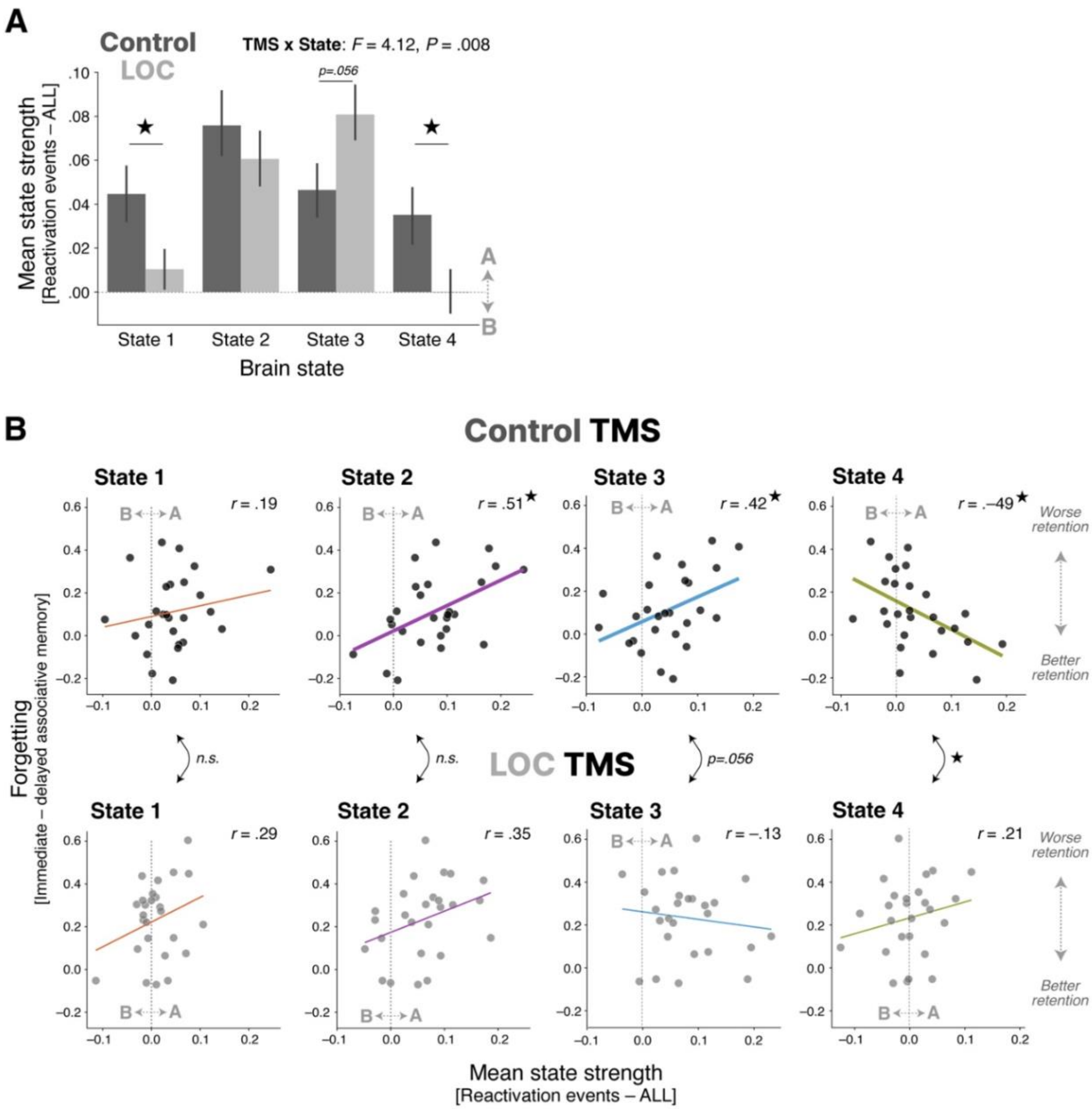

Figure S1. TMS effects on brain state coupling with reactivation and its link to memory. (A) Mean brain state strength during reactivation events, relative to mean strength across all time points, shown separately for Control TMS (dark gray) and LOC TMS (light gray) groups. TMS to LOC reduced State 1 and 4 strengths (expression of the 1A and 4A poles) during reactivation relative to Control TMS, and marginally increased State 3 strength (3A expression) during reactivation. Stars indicate significant pairwise comparisons ($p < .05$). (B) Scatter plots show the relationship between brain state strength during reactivation and forgetting (immediate minus delayed associative memory score) for each of the four brain states, plotted separately for the Control TMS (top) and LOC TMS (bottom) groups. Each data point denotes a single participant. Stars indicate significant correlations or interactions by TMS ($p < .05$).

bioRxiv preprint doi: https://doi.org/10.64898/2026.09.24.754200; this version posted September 30, 2026. The copyright holder for this preprint (which was not certified by peer review) is the author/funder, who has granted bioRxiv a license to display the preprint in perpetuity. It is made available under a CC-BY 4.0 International license.

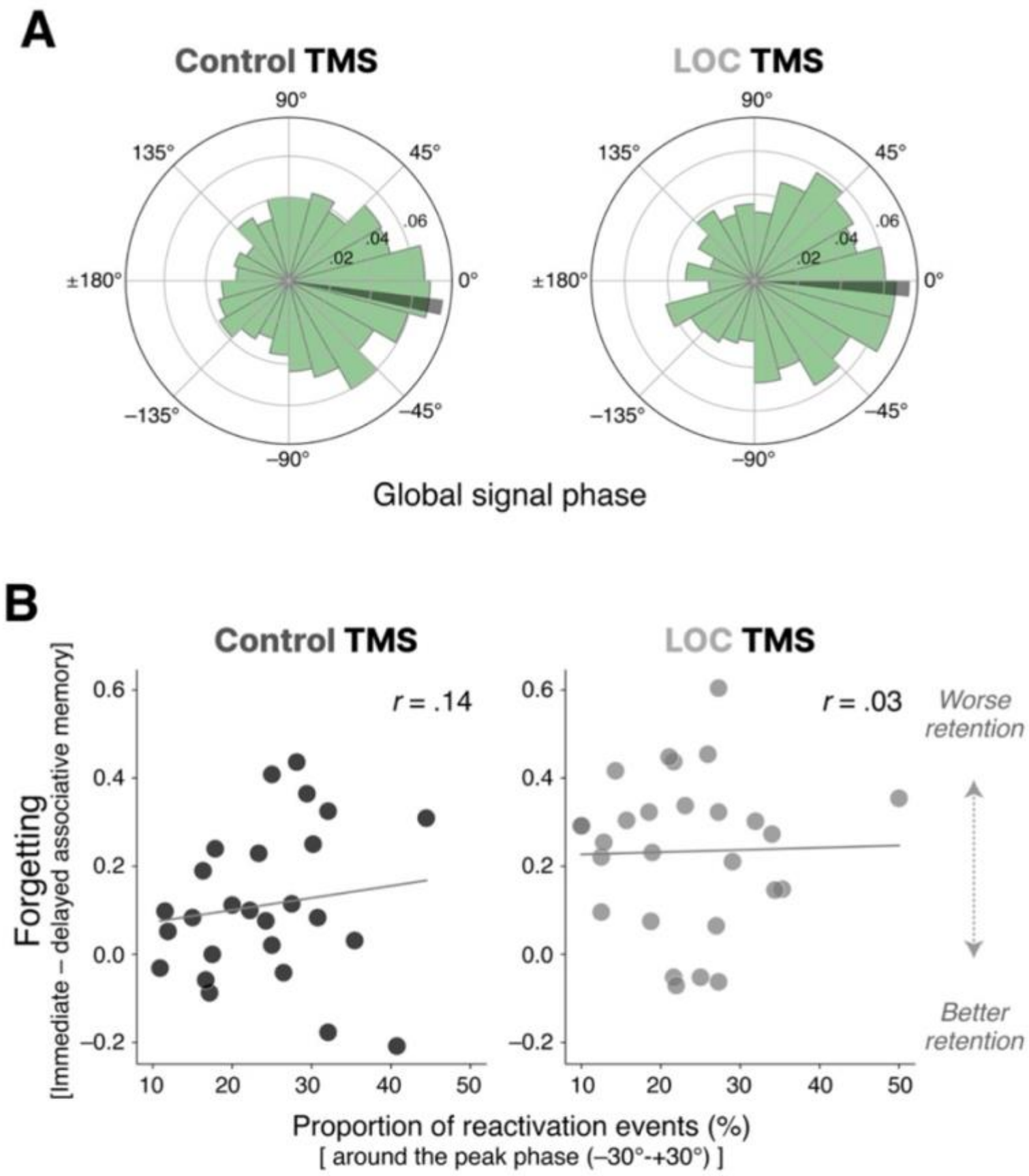

1598 **Figure S2. TMS effects on global signal (GS) phase coupling with reactivation and its link to memory.** (A) Distribution of GS phase of reactivation time points, aggregated across participants, shown separately for the Control TMS (left) and LOC TMS (right) groups. Reactivation events clustered around the peak GS phase (0°) in both groups, and this phase-locking did not differ by TMS site. (B) Scatter plots show the relationship between the proportion of reactivation events occurring near the peak GS phase (−30° to +30°) and forgetting (immediate − delayed associative memory score), plotted separately for the Control TMS (left) and LOC TMS (right) groups. Each data point denotes a single participant.

bioRxiv preprint doi: https://doi.org/10.64898/2026.09.24.754200; this version posted September 30, 2026. The copyright holder for this preprint (which was not certified by peer review) is the author/funder, who has granted bioRxiv a license to display the preprint in perpetuity. It is made available under a CC-BY 4.0 International license.

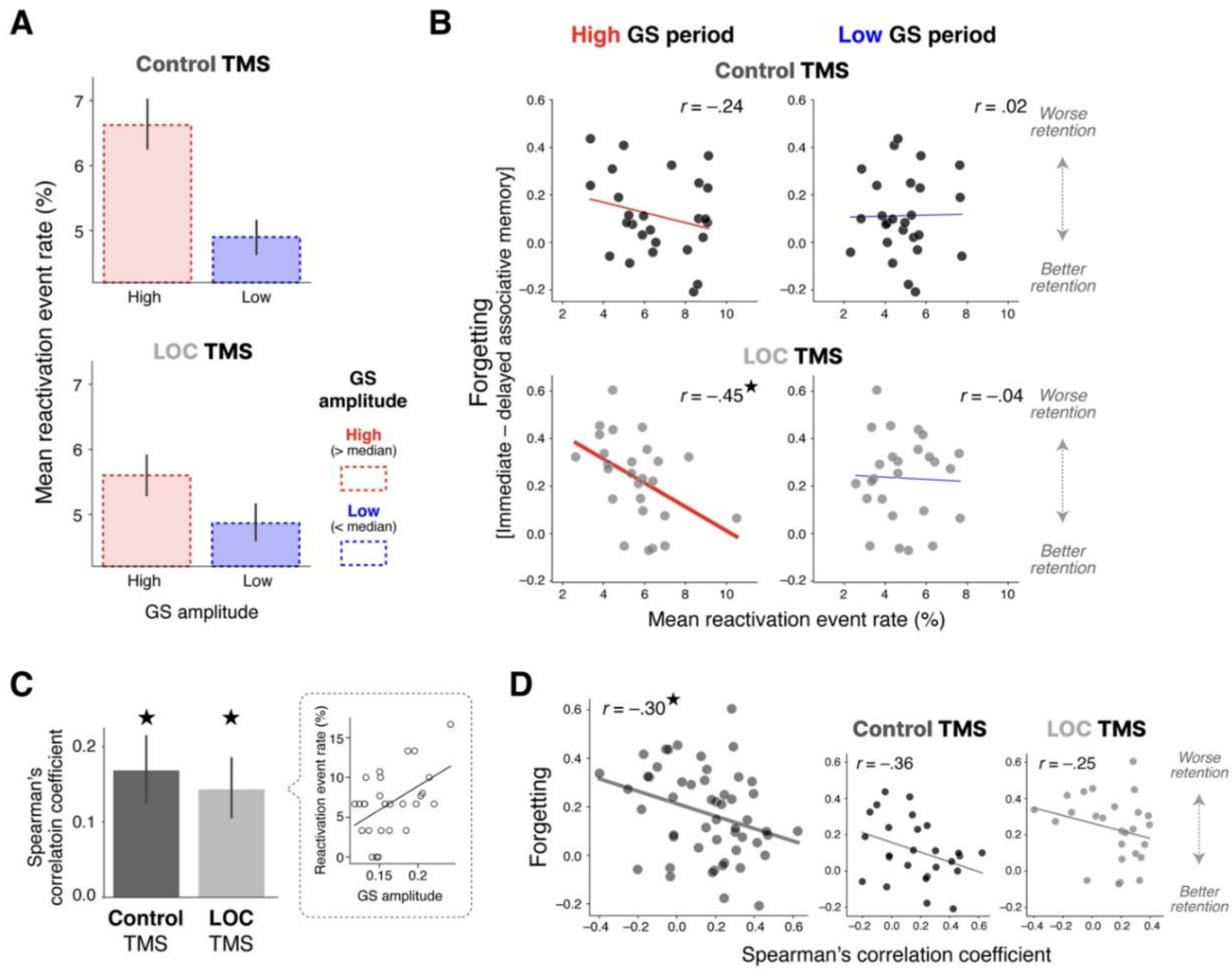

1599 **Figure S3. TMS effects on global signal (GS) amplitude coupling with reactivation and its link to memory.** (A) Mean reactivation event rate in high- versus low-amplitude GS windows, shown separately for the Control TMS (top) and LOC TMS (bottom) groups. (B) Scatter plots show the relationship between mean reactivation event rate and forgetting (immediate − delayed associative memory score) for high-GS (left) and low-GS (right) amplitude periods, plotted separately for the Control TMS (top) and LOC TMS (bottom) groups. Each data point denotes a single participant. (C) Spearman's correlation between GS amplitude and reactivation event rate across time windows within individual participants, shown separately for the Control TMS and LOC TMS groups (stars indicate correlations significantly above 0, $p < .05$). Inset shows an example scatter plot of reactivation event rate against GS amplitude across time windows for one participant. (D) Scatter plots show the relationship between the Spearman's correlation coefficient (GS amplitude–reactivation rate) and forgetting, across all participants (left) and separately for the Control TMS (middle) and LOC TMS (right) groups. Each data point denotes a single participant.

bioRxiv preprint doi: https://doi.org/10.64898/2026.09.24.754200; this version posted September 30, 2026. The copyright holder for this preprint (which was not certified by peer review) is the author/funder, who has granted bioRxiv a license to display the preprint in perpetuity. It is made available under a CC-BY 4.0 International license.

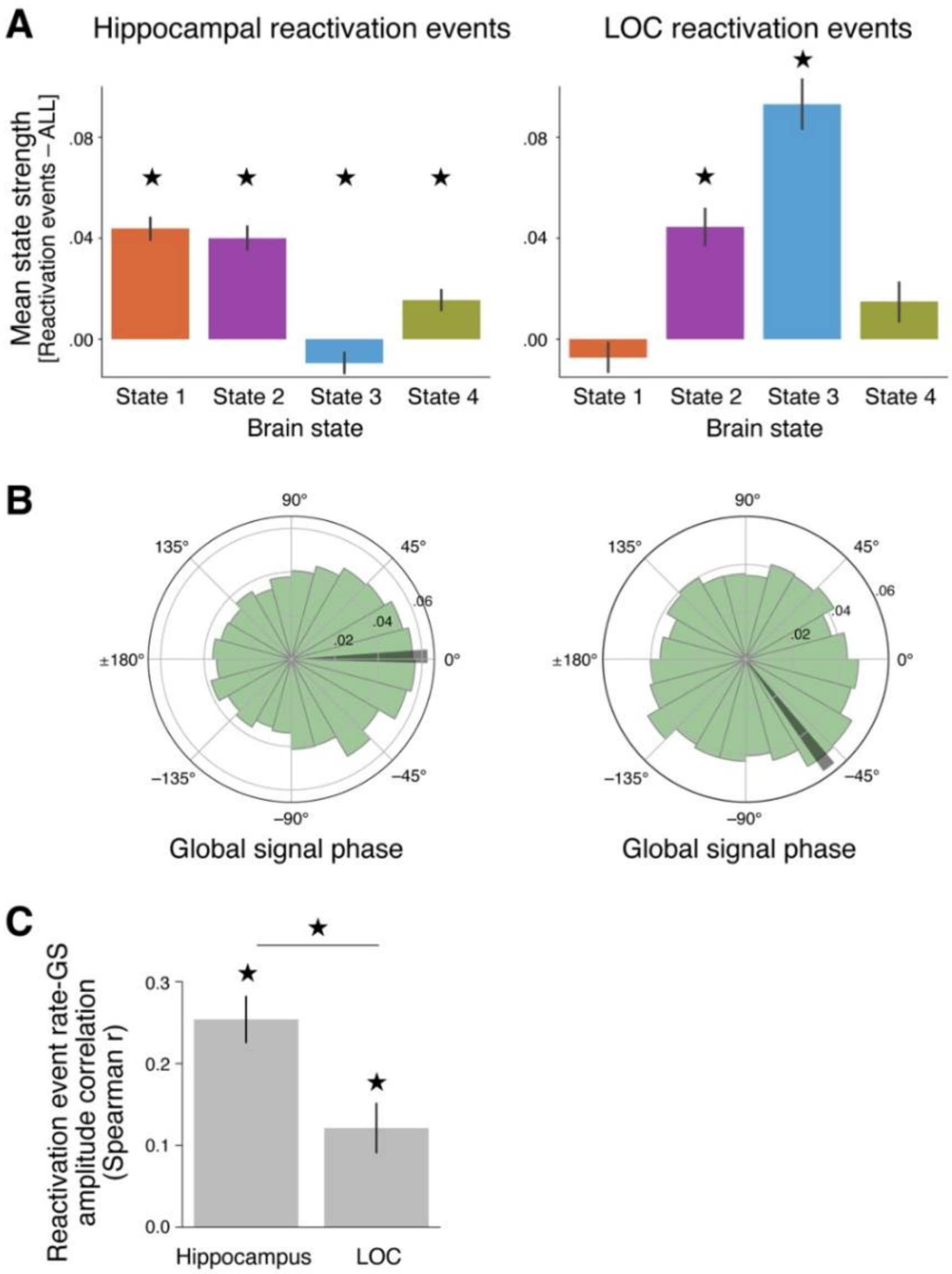

Figure S4. Region-specific coupling of hippocampal and LOC reactivation with brain states and global signal (GS) phase. (A) Mean brain state strength during reactivation events, relative to mean strength across all time points, for hippocampal (left) and LOC (right) reactivation events. Hippocampal reactivation was coupled with States 1, 2, and 4, and with the 'B' pole of State 3, whereas LOC reactivation was coupled with States 2 and 3 (biased toward the 'A' pole) but not reliably with State 1. Stars indicate FDR-corrected one-sample t-tests against 0 ($p < .05$). (B) Distribution of GS phase of reactivation time points for hippocampal (left) and LOC (right) reactivation events. Hippocampal reactivation clustered around the peak GS phase (0°), whereas LOC reactivation was linked to an earlier GS phase (peaking around −45°), indicating a phase offset between the two regions. (C) Spearman's correlation between reactivation event rate and GS amplitude across time windows within participants, shown separately for Hippocampal and LOC reactivation events (stars indicate correlations significantly above 0, and significant differences in correlation between the two regions, $p < .05$).
